# Systematic and Functional Identification of Small Non-Coding RNAs Associated with Excess Ammonium Stress in Cyanobacterium *Synechocystis* sp. PCC 6803

**DOI:** 10.3390/ijms27135667

**Published:** 2026-06-23

**Authors:** Ge Zhang, Taotao Zheng, Shiqi Lin, Siyu Chen, Gu Chen

**Affiliations:** School of Food Sciences and Engineering, South China University of Technology, 381 Wushan Road, Guangzhou 510640, China

**Keywords:** cyanobacterium, *Synechocystis*, sRNA, ammonium stress, ssr0692-as, PirA

## Abstract

Cyanobacteria, the only prokaryotic oxygenic phototrophs, rely on sophisticated regulatory networks, including those mediated by small RNAs (sRNAs) to cope with environmental fluctuations. Here, we delineate the sRNA landscape of *Synechocystis* sp. PCC 6803 under short- and long-term ammonium stress, revealing a significant proportion of antisense RNAs (asRNAs). Functional characterization identified three asRNAs (sll0312-as, sll0873-as, and slr1667-as) as key regulators of ammonium stress tolerance, implicating their targets (sll0312, sll0873, and slr1667) as new players in nitrogen fluctuation acclimation. The sll0944-as and sll1515-as were also identified, revealing an additional regulatory layer targeting known carbon/nitrogen metabolism regulators. Mechanistically, we characterized the ammonium-induced asRNA ssr0692-as, demonstrating that it represses *pirA* translation via direct 5′UTR interaction. This finding, integrated with the known role of the nitrogen limitation-responsive sRNA NsiR4 targeting the same region, supports a synergistic model wherein these two sRNAs precisely modulate PirA protein levels—and thus the downstream nitrogen flux—across varying nitrogen availability. Together, our findings expand the functional repertoire of cyanobacterial sRNAs and elucidate a dynamic post-transcriptional mechanism to fine-tune nitrogen metabolism in response to fluctuating nutrient conditions.

## 1. Introduction

Bacteria respond to environmental change quickly and efficiently through intricate multi-level regulatory networks operating at the transcriptional, post-transcriptional and post-translational levels. In general, the transcriptional level involves transcriptional factors as well as others, the post-translational level employs modifying or interacting proteins, while the post-transcriptional level might recruit regulatory small RNA (sRNA) as key player [1]. These 50–400 nt sRNA molecules bind mRNA targets completely or partially complementarily to modulate their stability and/or translation, leading to either activation (mRNA stabilization or translational enhancement) or repression (mRNA degradation or translational inhibition) [1,2]. sRNAs are integral to diverse stress responses and physiological processes, including carbon metabolism, iron homeostasis, environmental acclimation, and quorum sensing [1]. A comprehensive understanding of sRNA-mediated regulation is therefore essential for developing strategies to control bacterial growth—whether to combat pathogens or to optimize bacteria as biotechnological platforms [2,3,4]. Generally, trans-encoded sRNAs are usually, but not always, found in the intergenic region of genes, and form imperfect base-pairing with the mRNA targets, while cis-encoded antisense RNAs tend to form perfect double-stranded regions with the mRNA targets. The resulting base-pairing either enhances or represses the mRNA’s ability to be translated. Many Gram-negative bacteria use Hfq to chaperone the interaction between small non-coding RNA (sRNA) and mRNA to facilitate their annealing [5]; however, the Hfq homolog in cyanobacteria does not bind RNA, it functions as a regulator of cell motility in *Synechocystis* PCC 6803 instead of being an RNA chaperone [6,7,8]. While the homolog of another RNA chaperon common in bacteria ProQ is absent in *Synechocystis* PCC 6803, alternative RNA-binding protein candidates were suggested via gradient profiling by sequencing analyses, such as KhpA/B homologs Slr0287 and Slr1472 [8].

As the only prokaryotes capable of oxygenic photosynthesis, cyanobacteria rely on sophisticated regulatory networks, including sRNA-meditated mechanisms, to cope with environmental fluctuations [9]. Transcriptomic studies have revealed that both *cis*-encoded antisense sRNA (asRNA) and *trans*-encoded sRNA are prevalent in diverse cyanobacteria under normal and stress conditions, with examples reported in *Synechocystis* sp. PCC6803 (hereafter *Synechocystis* 6803) [10,11], *Nostoc* sp. PCC 7120 (*Nostoc* 7120) [12,13,14], and *Prochlorococcus* MED4 [15]. Despite the thousands of putative sRNAs identified in each cyanobacteria species, however, only about twenty have been functionally characterized to date. These characterized sRNAs are predominantly responsive to excessive light, nitrogen starvation, inorganic carbon limitation [16,17], iron starvation [18,19,20], short chain alcohol stress [21,22,23], and heterocyst differentiation [24,25].

In *Synechocystis* 6803, for instance, the light-induced asRNA PsbA2R targets the *psbA2* 5′UTR (Untranslated Region) to stabilize the mRNA and positively regulate expression [26]. The high light-induced sRNA PsrR1 post-transcriptionally controls several photosynthetic proteins and destabilizes *psaL* mRNA by promoting RNase E-mediated cleavage [27]. Another high light-induced asRNA, ThfR, regulates photosynthesis by stabilizing *thf1* mRNA, potentially through the protection of the RAUUW element at the RNase E cleavage site [28]. ApcZ, derived from the 3′ end of *apcABC-apcZ* operon, inversely regulates light harvesting and photoprotection by inhibiting orange carotenoid protein (OCP) expression under stress-free condition [29].

Several nitrogen starvation-responsive sRNAs have also been characterized. In *Synechocystis* 6803, the nitrogen deprivation-induced sRNA NsiR4 represses translation of *gifA* (encoding glutamine synthetase inactivating factor IF7) and *pirA* (encoding PII-interacting protein PirA) by targeting their 5′UTRs, positioning NsiR4 as a post-transcriptional regulator that facilitates rapid adaptation to nitrogen shifts [30,31]. Intriguingly, in *Nostoc* 7120, the nitrogen deprivation-induced sRNA, the NsiR4 homolog targets different mRNAs. It represses two Calvin cycle genes, *glpX* (encoding SBPase) and *pgk* (encoding phosphoglycerate kinase) via base-pairing their 5′UTR, thereby linking nitrogen response to CO_2_ assimilation [32]. More recently, the 3′UTR of the full *gifA* mRNA was shown to regulate glutamine synthetase (GlnA) in *Nostoc* 7120 via tail-to-tail overlap with the *glnA* gene [33].

Nitrogen is often a limiting nutrient in natural habitats. Paradoxically, the overuse of NO_3_^−^- and NH_4_^+^-based fertilizers in croplands, together with improper discharge of animal fecal and dye industrial effluent, have rendered nitrogen pollution a serious global concern, including the eutrophication of water reservoirs, soil contamination, and atmospheric pollution [34,35,36]. Although ammonium is the preferred nitrogen source [37], excess ammonium imposes toxicity and elicits stress responses in cyanobacteria [38,39]. Our previous work demonstrated that acclimation to ammonium stress is not merely the reverse of nitrogen limitation [40,41]; rather, it involves Site-2 proteases (S2P) Slr1821 [40] and Sll0528 [41], which coordinate carbon/nitrogen homeostasis primarily through transcriptional modulation. However, whether and how sRNAs contribute to the post-transcriptional regulation of ammonium stress responses remains unexplored.

Here, we systematically investigated the sRNA landscape of *Synechocystis* 6803 under short- and long- term ammonium stress. We identified three asRNAs critical for stress tolerance and mechanistically dissected one ammonium-induced asRNA, ssr0692-as, which inhibits *pirA* translation via 5′UTR interaction. Our findings expand the functional repertoire of cyanobacterial sRNAs and elucidate a dynamic post-transcriptional mechanism that fine-tunes nitrogen metabolism in response to fluctuating nutrient conditions.

## 2. Results and Discussion

### 2.1. The sRNA Landscape in Synechocystis 6803 Under Ammonium Stress

To investigate whether ammonium stress triggers differential expression of sRNAs, the sRNA landscape in *Synechocystis* 6803 was analyzed under short-term (30 min) and long-term (24 h) ammonium stress, which was induced by 30 mM NH_4_Cl. Samples without ammonium stress served as the background control (BC), while those treated with 30 mM NaCl were designated as an additional control to differentiate the effects of the ammonium ion from those of the chloride ion.

Approximately 2.8 Gb of raw data per sample were generated by UMI (unique molecular identifier) RNA sequencing [42,43]. Following adapter trimming and quality control, an average of 16,927,728 reads per sample were retained as clean data. These clean reads were then processed for UMI deduplication, yielding 13,037,696 reads per sample. Subsequent filtering removed ribosomal RNA (rRNA) reads, which accounted for 10.78% to 16.29% of the data. Finally, an average of 11,148,505 high-quality reads per sample were obtained for downstream analysis. Based on the experimentally anchored map of transcriptional start sites under different conditions and the sRNAs annotated in the same references [10,11], we identified 1070 antisense RNAs (asRNAs) and 318 known sRNAs; 43 novel sRNAs were predicted using sRNAscanner. Across all samples, a total of 1431 putative sRNAs were identified (Supplementary Table S1). The majority of identified sRNAs ranged from 25 to 250 nt in length. (Supplementary Figure S1). The reliability of the RNA sequencing data was demonstrated by sample correlation analysis (Supplementary Figure S2) and further validated by quantitative RT-PCR of representative sRNAs, which yielded a Pearson’s correlation coefficient of 0.8015 (Supplementary Figure S3).

**Figure 1:**
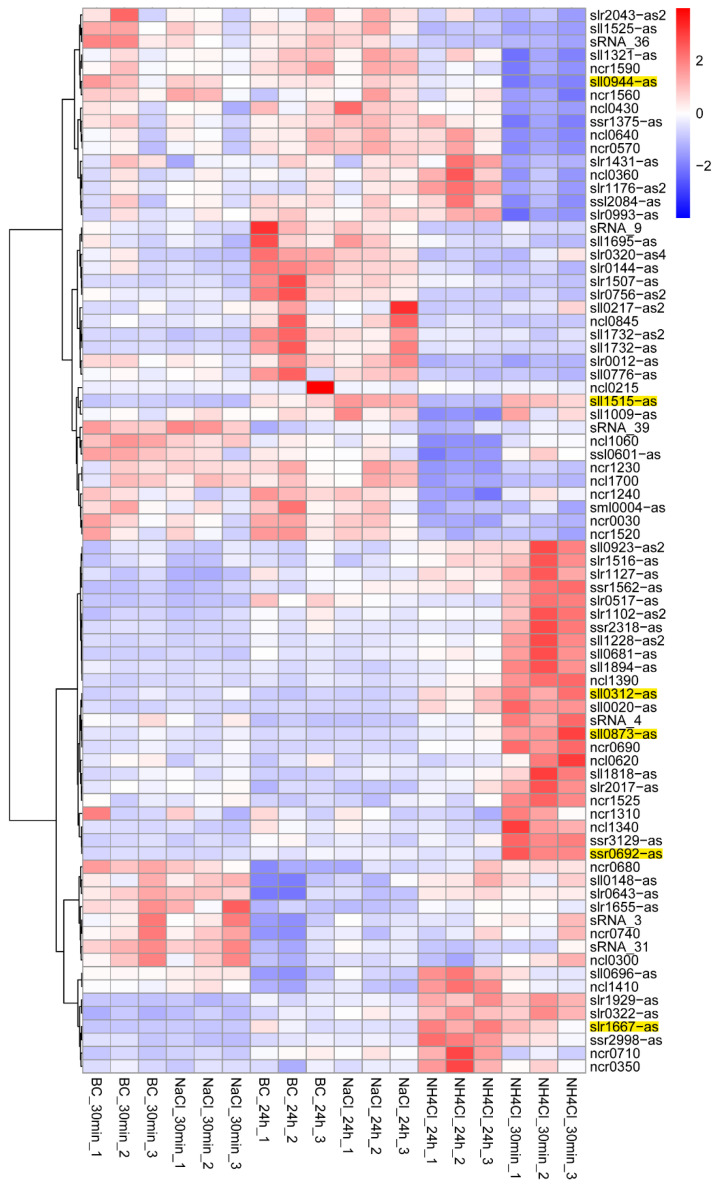
Hierarchical clustering of differentially expressed sRNAs. sRNAs were considered differentially expressed between groups using a threshold of FDR < 0.05 and |log_2_FC| ≥ 1. Expression levels (log_10_RPKM) are represented by a color scale, with red and blue indicating high and low abundance, respectively. The x-axis shows the samples, and the y-axis lists the sRNAs. Antisense sRNAs (asRNAs) selected for further characterization are highlighted in yellow. BC: background control.

Based on the expression profiles, eighty differentially expressed sRNAs were identified by comparing different groups using a threshold of FDR (False Discovery Rate) < 0.05 and |log_2_FC (Fold change)| ≥ 1 (Figure 1). Among these, 48 were antisense RNAs (asRNAs) targeting specific genes, 26 were known sRNAs, and six were novel sRNAs. Statistical analysis indicated that the differential expression was primarily driven by ammonium stress, with a secondary effect from cultivation time (24 h vs. 30 min). In contrast, NaCl treatment did not induce significant differential expression (Figure 1; Supplementary Table S1). Therefore, the differential expression observed in the NH_4_Cl groups compared to background control was attributable to the ammonium ion rather than the chloride ion.

**Figure 2:**
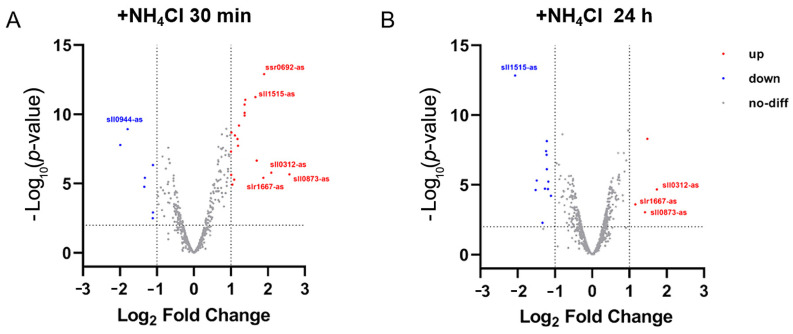
Differential expression of asRNAs in response to ammonium stress. Among the 1070 asRNAs, differentially expressed asRNAs were identified against background control (FDR < 0.05, |log_2_FC| ≥ 1) under short-term (30 min; (**A**)) and long-term (24 h; (**B**)) treatment with 30 mM NH_4_Cl. Each dot represents an asRNA, with red and blue colors denoting up- and down-regulation, respectively. Those asRNAs chosen for further characterization are annotated.

Our analysis revealed, for the first time, that ammonium stress triggers the differential expression of dozens of sRNAs in *Synechocystis* 6803, with asRNAs constituting approximately 60% of these (Figure 1). We then focused on the ammonium-responsive asRNAs under short-term and long-term stress, which accounted for 3.4% of all identified asRNAs (Figure 2).

### 2.2. Characterization of Ammonium-Responsive asRNAs

**Figure 3:**
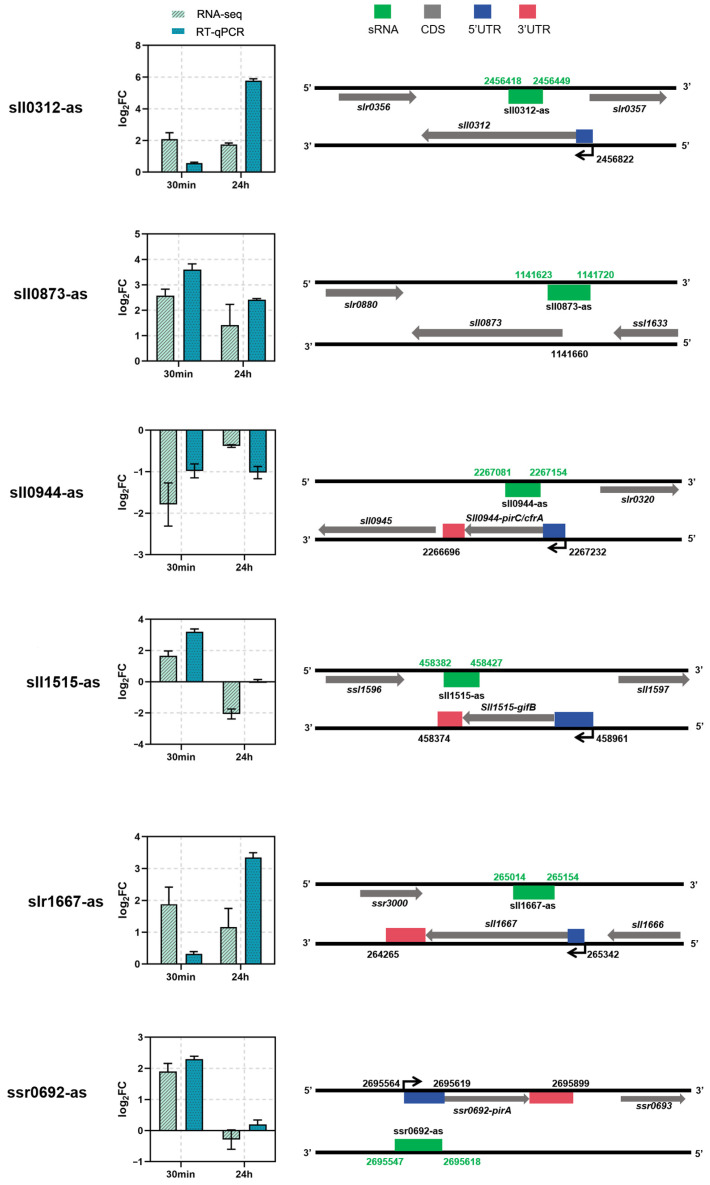
Differential expression and the localization of selected ammonium-responsive asRNA. The left columns display the relative abundance of asRNA in sRNA-sequencing data and RT-qPCR upon short-term (30 min) or long-term (24 h) ammonium stress induced by 30 mM NH_4_Cl. Samples without ammonium stress served as the background control (BC). The log2 Fold change of ammonium stress verse BC was used as Y axis. The right columns indicate the localization of asRNA relative to its target mRNA. The transcript start sites were experimental anchored in Ref, while the 3′ end was based on the longest transcript read [10,11].

As cis-encoded elements, asRNAs can positively or negatively regulate their target mRNAs by base-pairing to the 5′UTR, 3′UTR, or coding region [16,17,20,26,28,44,45]. To elucidate their functions, we selected six asRNAs significantly responsive to short-term and/or long-term ammonium stress for further characterization (Figure 3). Among these, sll0873-as and ssr0692-as target the 5′UTR, sll1515-as targets the 3′UTR, while sll0312-as, slr1667-as, and sll0944-as target the coding regions of their respective mRNAs (Figure 3). RT-PCR verification of the expression of these asRNAs generally matches their induction or repression obtained in RNA-sequencing data (Figure 3).

**Figure 4:**
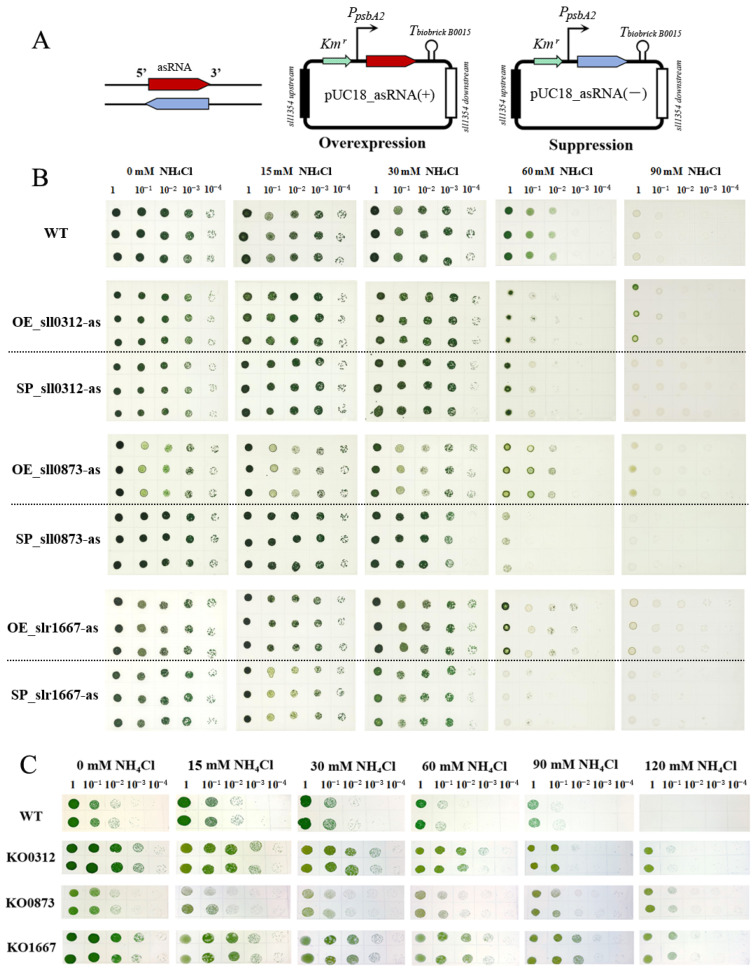
Functional analysis of ammonium-responsive asRNAs. (**A**) Strains were constructed to overexpress the asRNA or its antisense sequence from the psbA2 promoter respectively, as overexpression strains (OE) or suppression strain (SP). (**B**) Ammonium stress tolerance of overexpression (OE) or suppression (SP) strain of ammonium-responsive asRNAs. Suppression strain of three asRNAs, sll0312-as, sll0873-as, and slr1667-as, had decreased ammonium stress tolerance compared with the overexpression strain. Ammonium sensitivity was assessed using a drop-plate assay with at least three biological replicates per strain. Shown is a representative result. Liquid cultures were adjusted to OD_730_ = 1.0, serially diluted tenfold, and spotted onto BG-11 agar plates containing increasing concentrations (15 to 90 mM) of ammonium chloride (NH_4_Cl). (**C**) Ammonium stress tolerance of knockout strain (KO) of sll0312, sll0873, and slr1667.

Stable overexpression or suppression strains for these six asRNAs in *Synechocystis* 6803 were constructed by expressing the asRNA or its antisense sequence, respectively, under the control of the psbA2 promoter (Figure 4A). This light-inducible promoter had been confirmed to drive strong constitutive expression of the following gene under the culture condition used in our lab with constant illumination (30 μE·m^−2^·s^−1^) [46]. Both overexpression (OE) and suppression strains (SP) were generated through the integration of fragment into the same *sll1354* loci of the *Synechocystis* 6803 genome, and carrying the same kanamycin resistance gene as selection marker. Thus, the phenotype difference between OE and SP strains could indicate the distinct effect of expression of asRNA or its antisense sequence.

Under normal growth conditions, all strains exhibited growth curves and chlorophyll accumulation similar to the wild type (WT) over five-day cultivation (Supplementary Figure S4). However, distinct differences in their responses to ammonium stress were found in some asRNAs (Figure 4B).

### 2.3. Modulation of Specific asRNA Expression Affects Tolerance to Ammonium Stress

Ammonium tolerance was assessed using drop-plate assays with increasing concentrations (15–90 mM) of ammonium chloride. Intriguingly, for three asRNAs—sll0312-as, sll0873-as, and slr1667-as—the overexpression and suppression strains exhibited different ammonium sensitivity phenotypes (Figure 4B, Supplementary Figure S5A–C). The suppression of these three asRNAs remarkably impaired the ammonium tolerance, whereas the overexpression strains indicated higher stress tolerance than the suppression strain. In contrast, no significant difference in ammonium tolerance was found between the overexpression and suppression strains of sll0944-as, sll1515-as, and ssr0692-as (Supplementary Figure S5D).

The notable reduced ammonium stress tolerance in their suppression strains suggest that sll0312-as, sll0873-as, and slr1667-as are critical regulators of their target mRNAs and that the corresponding target proteins might play essential roles during acclimation to ammonium stress. For the remaining three asRNAs, the absence of clear phenotypic difference between the overexpression and suppression strains suggests their roles may be redundant, or only part of a regulatory system that was not decisive under our experimental condition.

Notably, the three functionally validated asRNAs, sll0312-as, sll0873-as, and slr1667-as, shared a consistent upregulation pattern under both short-term and long-term ammonium stress (Figure 3), aligning perfectly with the reduced tolerance observed in their suppression strains (Figure 4B). Conversely, the other asRNAs exhibited more transient or variable expression patterns upon ammonium stress. The sll1515-as and ssr0692-as were induced only in short-term stress; while sll0944-as was downregulated in ammonium stress. This diverse expression kinetics suggests that, in addition to asRNA-mediated regulation, their mRNA targets are likely under complex, multi-layered control.

We further generated the knockout strains of *sll0312*, *sll0873*, and *slr1667*. Enhanced ammonium stress tolerance was observed in their knockout strains, compared with WT (Figure 4C). Thus, these three asRNAs were found to negatively regulate their target gene expression during ammonium acclimation, and these three target genes might play negative roles in the ammonium stress acclimation. The *sll0312* gene is predicted to encode a permease component of a peptide/nickel transporter. A potential role for Sll0312 as an ammonium transporter or sensor represents an intriguing direction for future research. Sll0873 was previously annotated as a carboxynorspermidine decarboxylase [47], but it was recently re-characterized as a carboxyaminopropylagmatine (CAPA) decarboxylase, which converts CAPA into aminopropylagmatine (APA) in the spermidine biosynthesis pathway [48]. The molecular functions of CAPA, APA, and spermidine in the context of ammonium stress and nitrogen limitation tolerance merit further investigation. Slr1667-as targets the *slr1667* gene, which encodes a secreted protein (Slr1667) implicated in cell surface assembly [49,50]. The link between this putative surface component and ammonium stress signaling remains unclear and highlights a novel avenue for mechanistic exploration in the future.

Another two asRNA targets, Sll0944 and Sll1515, were both known as important components involved in adaption to nitrogen deficiency. Sll0944 (PirC/CfrA) is upregulated upon nitrogen deprivation, and is liberated from PII sequestration to bind and inhibit activity of phosphoglycerate mutase (PGAM)—the enzyme that deviates newly fixed CO_2_ toward lower glycolysis [51,52]. Sll1515 (GifB) is the glutamine synthetase inactivating factor IF17, and its transcription is repressed by NtcA [53] and controlled through glutamine riboswitch [54]. Here, our study revealed sll0944-as and sll1515-as as ammonium responsive asRNA, thus suggesting an additional delicate layer of control on these known carbon/nitrogen metabolism regulators.

### 2.4. Ammonium Responsive sRNA ssr0692-as Represses pirA Translation via Interaction with Its 5′UTR

**Figure 5:**
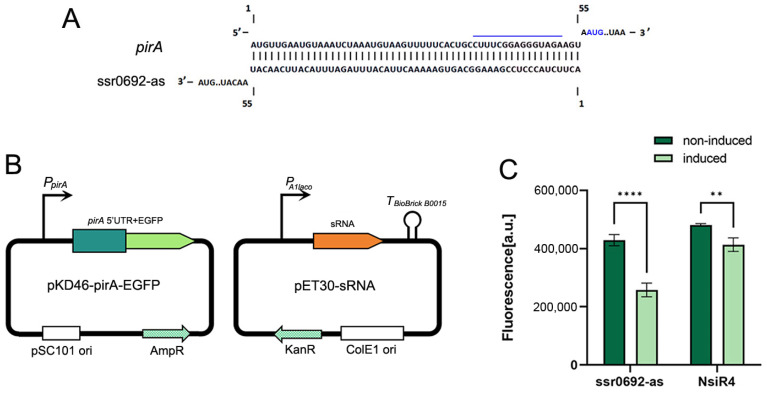
Reporter assays verify the interaction between ssr0692-as and the *pirA* 5′UTR. (**A**) The ssr0692-as sequence complementarily covers the 5′UTR of *pirA*, spanning the ribosome binding site upstream of the AUG start codon highlighted in blue. The *pirA* numbering is relative to the transcriptional start site (TSS, +1). The overline above *pirA* denotes the known NsiR4 binding motif. (**B**) Design of the dual-plasmid co-expression system. The full-length *pirA* 5′UTR and its first 60 nt were fused in frame with EGFP coding sequence and cloned into the low-copy number pKD46 vector (replication origin pSC101). The sRNA, ssr0692-as or NsiR4, was expressed from the IPTG-inducible promoter PA1lacO-1 in the high-copy number pET30 vector (ColE1 origin). (**C**) GFP fluorescence was significantly reduced upon IPTG-induced sRNA expression. The repression was more pronounced with ssr0692-as than with NsiR4 when they were co-expressed with the *pirA* 5′UTR-EGFP reporter. Data represent the mean ± SD of three independent experiments using Top10 *E. coli* cells harboring both plasmids depicted in (**B**). ****, *p* < 0.0001; **, *p* < 0.01.

We selected ssr0692-as for further analysis of its regulatory function. First, its target gene, *ssr0692*, encodes PirA (PII interacting regulator of arginine synthesis), a key integrator determining nitrogen flux into storage compounds [55]. Second, PirA is known to be post-transcriptionally regulated by another sRNA, NsiR4 [31]. Sequence alignment revealed that ssr0692-as complementarily covers the *pirA* 5′UTR, spanning positions -2 to -56 relative to the start codon (Figure 5A). Such interaction is predicted to occlude the ribosome binding site, thereby inhibiting *pirA* translation.

We first verified this RNA-RNA interaction using heterologous reporter assays in *E. coli.* The *pirA* 5′UTR and its first 60 nt coding region were fused in frame with *egfp* gene and co-expressed with ssr0692-as. In two independent plasmid combinations, (using either medium- or low-copy number reporter vector), IPTG-induced expression of ssr0692-as significantly reduced GFP fluorescence (Figure 5 and Supplementary Figure S6). As a comparative control, co-expression of NsiR4 in the same system also diminished fluorescence notably, albeit to a lesser extent (Figure 5C). A shorter version of NsiR4 lacking of first seven nucleotides was found to have no significant repression effect and thus served as the negative control (Supplementary Figure S6).

**Figure 6:**
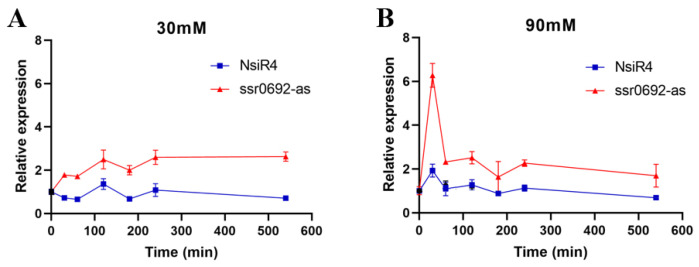
Temporal expression profiles of ssr0692-as and NsiR4 during ammonium stress. The accumulation kinetics of ssr0692-as and NsiR4 transcripts in response to treatment with 30 mM (**A**) or 90 mM (**B**) NH_4_Cl were determined by quantitative RT-PCR over 9 h. Data represent the mean ± SD of three independent experiments using *rnpB* as internal control. Expression level at time zero was set as 1.

We next quantified the transcript accumulation kinetics of ssr0692-as and NsiR4 under ammonium stress via quantitative RT-PCR. Three independent genome-wide mappings of the transcriptional start sites (TSS) in *Synechocystis* 6803 under different conditions all anchored the TSS of ssr0692-as to the same point, 2,695,618 [10,11,56]. While the 3′end was determined based on the longest transcript read [10,11]. RT-PCR using primers extending into the upstream or downstream of ssr0692-as confirmed that ssr0692-as is not simply an extension of surrounding mRNA (Supplementary Figure S6C,D). Specific primer for ssr0692-as, rather than the random primer, was used in reverse transcription to differentiate the transcript of ssr0692-as and *pirA*. Over a 9 h exposure to 30 mM or 90 mM NH_4_Cl, ssr0692-as and NsiR4 exhibited distinct expression profiles (Figure 6). Intriguingly, ssr0692-as transcript levels nearly doubled under 30 mM NH_4_Cl throughout the time course, whereas NsiR4 levels showed no notable change. Under 90 mM NH_4_Cl, ssr0692-as transcript remarkably increased at 30 min and then remained elevated at about two-fold throughout 9 h; however, NsiR4 was transiently upregulated at 30 min and quickly returned to baseline after that. These data suggest that ssr0692-as, as an ammonium-responsive sRNA, might serve as a primary sRNA to fine tune the translation of PirA upon ammonium stress.

**Figure 7:**
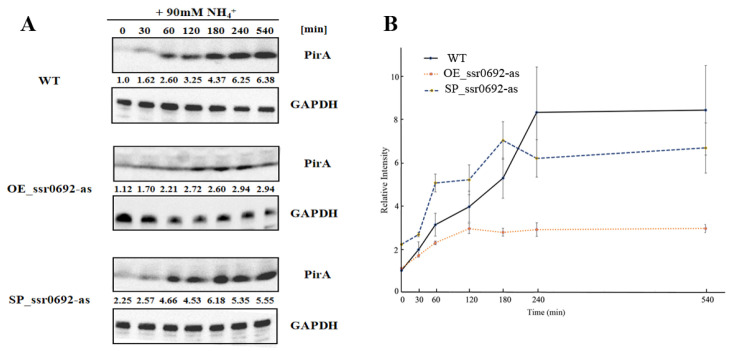
PirA abundance in ssr0692-as overexpression and suppression strains. (**A**) Representative Western blots of PirA (using anti-PirA antibodies) and the loading control GAPDH in different strains treated with 90 mM NH_4_Cl for the indicated durations. (**B**) Quantitative analysis of PirA abundance in (**A**). The signal intensities of PirA in (**A**) were quantified, normalized to the corresponding GAPDH signal, and presented relative to the wild type (WT) at time zero (set as 1).

To assess the physiological relevance, we examined PirA protein dynamics in *Synechocystis* 6803 wild-type and ssr0692-as overexpression or suppression strains under 90 mM NH_4_Cl stress. PirA abundance increased sharply in all strains following ammonium shock (Figure 7), similar to its upregulation by 10 mM NH_4_Cl in previous reports [31,55]. However, the induction profiles differed in three strains. Compared to the wild type, the overexpression strain accumulated less PirA after 60 min, reaching only about half the WT level at 4–9 h. Conversely, the suppression strain exhibited a slightly early induction following ammonium shock. These results demonstrate that ssr0692-as inhibits PirA accumulation in vivo.

In summary, heterologous reporter assays confirmed that ssr0692-as directly targets the *pirA* 5′UTR and mediates stronger repression than NsiR4 (Figure 5). Western blot analysis in *Synechocystis* 6803 conclusively demonstrated that ssr0692-as negatively regulates PirA protein abundance in vivo (Figure 7). This regulation is physiologically relevant. Although both sRNAs converge on the *pirA* 5′UTR (Figure 5), ssr0692-as is more robustly induced by ammonium stress (Figure 6), whereas NsiR4 is primarily responsive to nitrogen limitation [30]. Their differential induction patterns suggest a model wherein these two sRNAs act synergistically to adjust PirA levels across varying nitrogen conditions. Notably, the reduction in PirA observed in the ssr0692-as suppression strain at 4–9 h (Figure 7) may be due to the regulation by other factors.

The bacterial transcript’ 5′UTR is usually the target of precise post-transcriptional regulation. Functionally, PirA competes with NAGK for binding to the PII protein, thereby inhibiting PII-mediated activation of NAGK and downregulating arginine synthesis [55]. PirA transcripts accumulate under ammonium upshift mainly due to relief from NtcA repression, and its translation is known previously to be fine-tuned by NsiR4 [31,55]. Our work here reveals an additional post-transcriptional layer of control. The identification of ssr0692-as as an ammonium-induced inhibitor of *pirA* translation establishes a precise mechanism for the dynamic modulation of metabolic flux in response to nitrogen availability. Also, differently from the NtcA transcriptional control of NsiR4 and *pirA*, no NtcA binding sites similar to GTA/N8/TAC [57] could be found across the ssr0692-as promoter region. Thus it is suggested that seeking the transcriptional regulator of ssr0692-as might reveal transcriptional regulation features different from NtcA.

## 3. Materials and Methods

### 3.1. Strains and Growth Conditions

*Synechocystis* sp. PCC 6803 GT-G strain, derived from ATCC 27184, was used as the wild type (WT) [58]. Cells were cultivated in BG11 medium [59] at pH 7.5, buffered with 20 mM HEPES, at 30 °C under ambient CO_2_ and constant illumination (30 μE·m^−2^·s^−1^). Mutant strains were cultivated in the presence of 50 μg·mL^−1^ kanamycin. For the background control (BC) cultivated in BG11, the ammonium concentration was 0.037 mM from ferric ammonium citrate. For ammonium stress, the cells were cultured in BG11 medium supplemented with various concentrations of NH_4_Cl.

### 3.2. UMI RNA Sequencing

Wild-type *Synechocystis* 6803 was cultured in 30 mM NH_4_Cl or 30 mM NaCl for 30 min (short-term) or 24 h (long-term). Samples without stress served as the background control (BC). Three replicates were included in each group. Total RNAs were extracted via TRIzol™ Reagent (Invitrogen, Carlsbad, CA, USA). The RNA sample was quantified by Qubit Fluorometer (Thermo Fisher Scientific, Waltham, MA, USA) and the integrity of the RNA was assessed by Bioanalyzer 2100 (Agilent, Santa Clara, CA, USA). The cDNA library was constructed by Small RNA Library Preparation Kit (Illumina, San Diego, CA, USA) including unique molecular identifiers (UMIs) [43], and subjected to sequencing at Illumina NovaSeq (Kangce SeqHealth, Wuhan, China). Raw data were filtered by software Trimmomatic (v 0.32) to remove adapter and low-quality reads to get clean reads. The clean data was then subjected to UMI deduplication to remove the duplication generated through PCR amplification. The deduplicated data was filtered to remove the rRNA reads. Then the data were mapped to the reference genome [58,60] by software Bowtie, as well as compared with the sRNA dataset from references [10,11] to identify known sRNA. Novel sRNAs were predicted through sRNAscanner (v 1.0). RPKM normalization was performed using Partek Genomics Suite (v 7.0). The read count for each sRNA was normalized by the total number of mapped reads (per million) and by the sRNA length (per kilobase). Differentially expressed sRNAs were visualized in a heatmap generated by hierarchical clustering using correlation distance and average linkage. The raw data was deposited at: https://www.ncbi.nlm.nih.gov/sra/PRJNA1423476 (assessed on 18 June 2026).

### 3.3. Construction of asRNA Overexpression and Suppression Strains

Based on the pUC18 vector, asRNA or its antisense sequence was cloned under the control of the psbA2 promoter and appropriate terminator (Figure 4 and Supplementary Table S2). Through natural transformation and homologous double recombination [41,61,62], asRNA or its antisense sequence and kanamycin resistance gene were integrated into *sll1354* loci of *Synechocystis* 6803 genome [63].

### 3.4. Construction of Knockout Strains of Sll0312, Sll0873, and Slr1667

The upstream and downstream regions of specific gene were cloned into pUC19 vector, between which the kanamycin resistance gene was inserted to generate the knockout strain through natural transformation and homologous double recombination. The primers used were listed in Supplementary Table S3.

### 3.5. Reporter Assays for Verification of asRNA:mRNA Interaction

The full-length *pirA* 5′UTR and its first 60 nt were fused in frame with EGFP coding sequence and cloned into the medium-copy number vector p15A with p15A ori derived from pKT25 plasmid or low-copy number vector pKD46 with pSC101 ori (Figure 5, Figure S6 and Supplementary Table S4). The sRNA, ssr0692-as or NsiR4, was expressed from the IPTG-inducible promoter PA1lacO [64] in the high-copy number vector pET30 with ColE1 ori (Figure 5 and Supplementary Table S4). The co-expression of different plasmid combination in *E. coli* (Top10) was selected via 50 μg·mL^−1^ kanamycin and 100 μg·mL^−1^ ampicillin. For every plasmid combination, eight independent clones were picked to inoculate 10 mL LB medium with the corresponding antibiotics at 30 °C for 5 h. Then, 100 μL pre-culture was used to inoculate 2 mL LB medium, one with 1 mM IPTG, and another one without IPTG at 30 °C for 10 h. Fluorescence was measured in CytoFLEX flow cytometer (Beckman Coulter, Brea, CA, USA) in FITC 488 nm with FSC ≥ 10,000, SSC ≥ 10,000 in 50,000 cells.

### 3.6. Quantitative RT-PCR

Total RNA was extracted as previously described [41]. Residual DNA was removed using DNase and verified by PCR amplification without reverse transcription. The *rnpB* gene encoding the RNA subunit of RNase P served as an internal control. The primers used are listed in Supplementary Table S5. Specific primers for sRNA, rather than the random primer, were used in reverse transcription to differentiate the transcript of asRNA and their target mRNA. At least three independent biological replicates for each sample and three technical repeats per biological replicate were analyzed. The relative expression level of the gene was calculated using the 2^−ΔΔCt^ method.

### 3.7. Western Blot Analysis

Crude protein extracts were prepared from 50 mL culture after centrifugation, PBS wash, re-suspension in 500 μL lysis buffer (50 mM HEPES-NaOH, 50 mM KCl, 1 mM PMSF), and disruption with ceramic beads as previously described [41]. Subsequently, proteins were separated in 15% SDS-PAGE and transmitted to PVDF membranes. Antibodies against PirA and the loading control GAPDH were diluted 1:5000 (anti-PirA) or 1:1000 (anti-GAPDH). Blocking, incubation and washing of membranes were performed as previously described [41].

## 4. Conclusions

Through sRNA-sequencing analysis of *Synechocystis* 6803, we revealed that ammonium stress triggers the differential expression of dozens of sRNAs, approximately 60% of which are antisense RNA (asRNA). Functional characterization revealed three asRNAs, sll0312-as, sll0873-as, and slr1667-as, as critical for acclimation to ammonium stress, with their suppression strains exhibiting reduced tolerance. This suggests that their target proteins, Sll0312, Sll0873, and Slr1667 as new participants in nitrogen fluctuation acclimation. We identified sll0944-as and sll1515-as as ammonium responsive asRNAs, adding a delicate layer of regulatory control to known carbon/nitrogen metabolism regulators. Mechanistically, we deciphered the regulatory function of an ammonium-induced asRNA, ssr0692-as. Reporter assays and Western blot analysis confirmed that ssr0692-as represses *pirA* translation by directly targeting its 5′UTR. Notably, while both ssr0692-as and the known sRNA NsiR4 target the same region, they exhibit distinct expression patterns: ssr0692-as is more robustly induced by ammonium, whereas NsiR4 is primarily responsive to nitrogen limitation. This suggests a synergistic model wherein these two sRNAs dynamically adjust PirA levels across a spectrum of nitrogen availability. Collectively, our study maps the sRNA landscape under ammonium stress and defines a novel post-transcriptional regulator on critical node-PirA mediated nitrogen flux. The discovery of ssr0692-as, as well as other ammonium responsive sRNAs, adds a precise layer of control to fine-tuning nitrogen homeostasis in response to nitrogen fluctuations.

## Data Availability

The data underlying this article are available in the article and in its online Supplementary Materials.

## Supplementary Materials

The following supporting information can be downloaded at: https://www.mdpi.com/article/10.3390/ijms27135667/s1.
