# Supplementary material for: Systematic and Functional Identification of Small Non-Coding RNAs Associated with Excess Ammonium Stress in Cyanobacterium Synechocystis sp. PCC 6803
Source: Int J Mol Sci. 2026 Jun 23;27(13):5667. doi: 10.3390/ijms27135667 (PMC13362535; doi:10.3390/ijms27135667)
Supplement: Supplementary file 1 [file ijms-27-05667-s001.zip › supplementary figures-clean version.pdf]

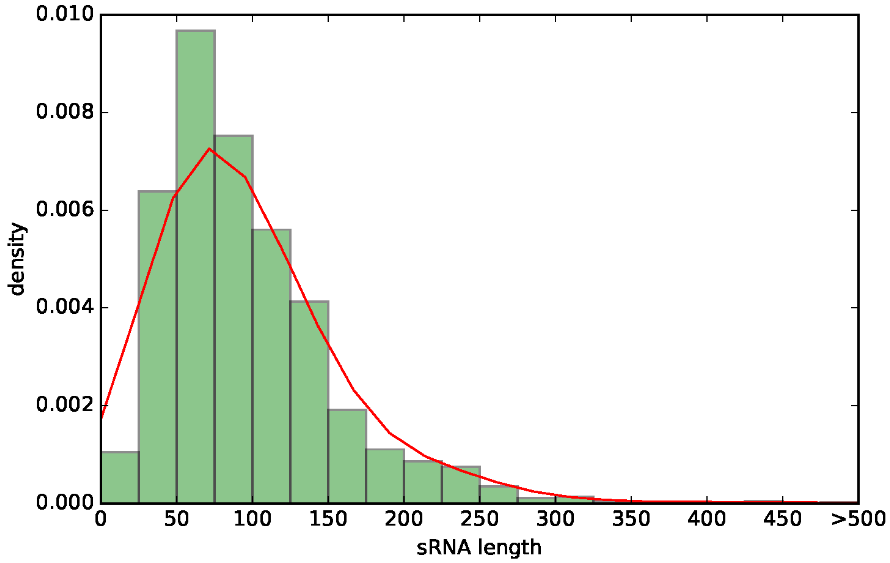

Supplementary Figure S1. Length distribution of sRNAs identified. For the 1,431 sRNAs identified, their lengths in nucleotides were analyzed to display the length distribution.

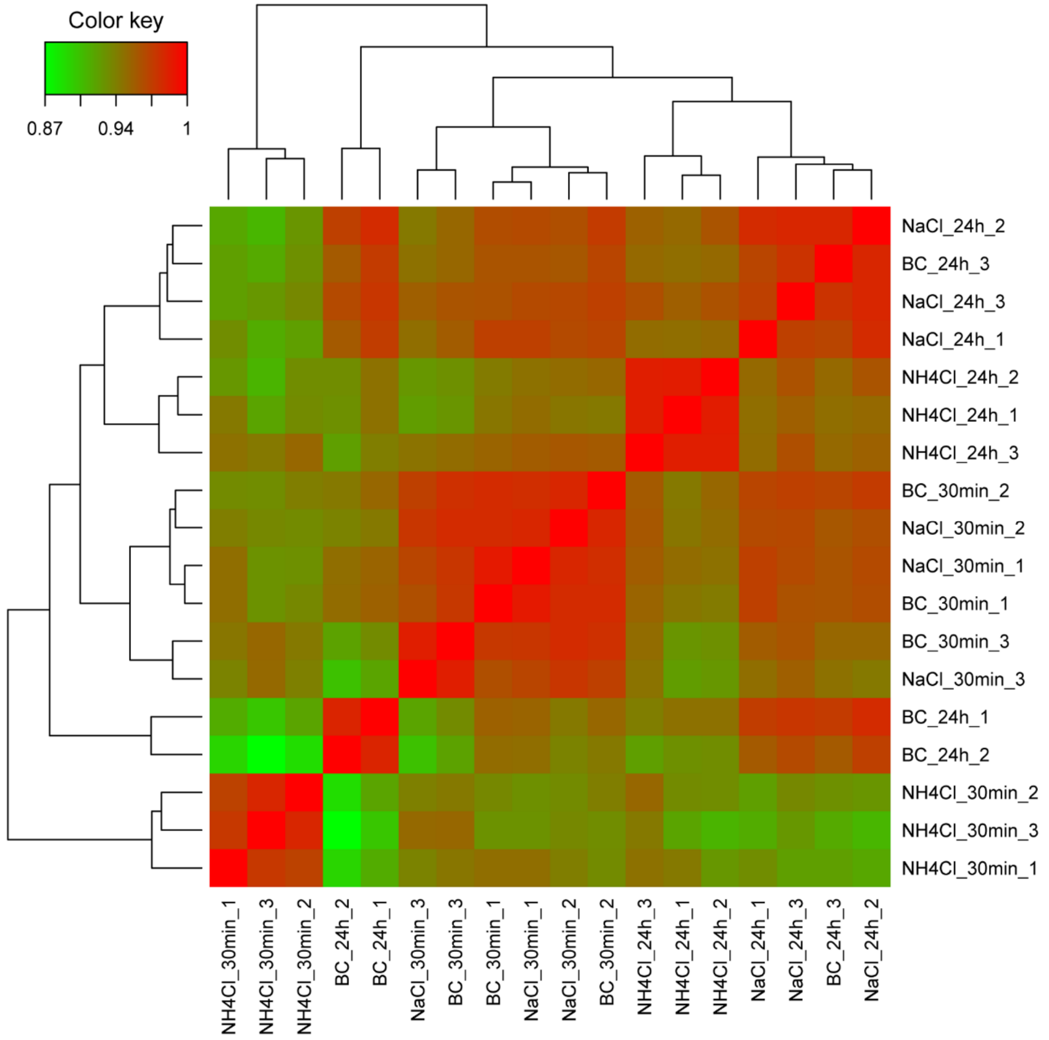

Supplementary Figure S2. Sample correlation heatmap of sRNA-sequencing data. The green to red color scale indicates the low to high correlation between samples based on their sRNA expression level.

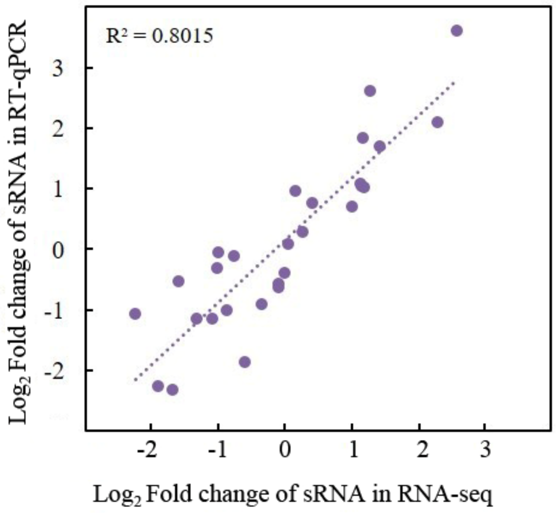

Supplementary Figure S3. RT-qPCR verification of sRNA sequencing data. The sRNA expression level of seven representative sRNAs in samples with or without ammonium stress for 30 min or 24 hour was quantified through RT-qPCR. The log2 fold change of expression level derived from UMI RNA sequencing and RT-qPCR in comparison pairs were used to generate the figure.

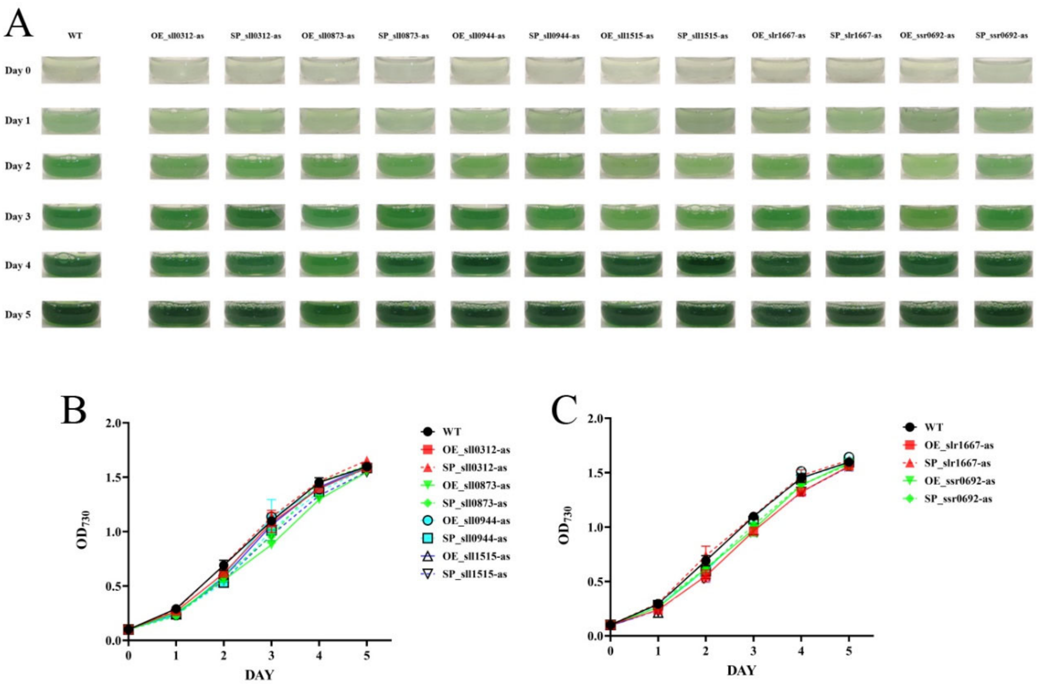

Supplementary Figure S4. The growth status of asRNA overexpression or suppression strains under normal growth condition. The asRNA overexpression or suppression line did not show significant difference with wild type (WT) under normal growth condition, which was indicated by similar degree of greening (A) and similar growth curve (B, C).

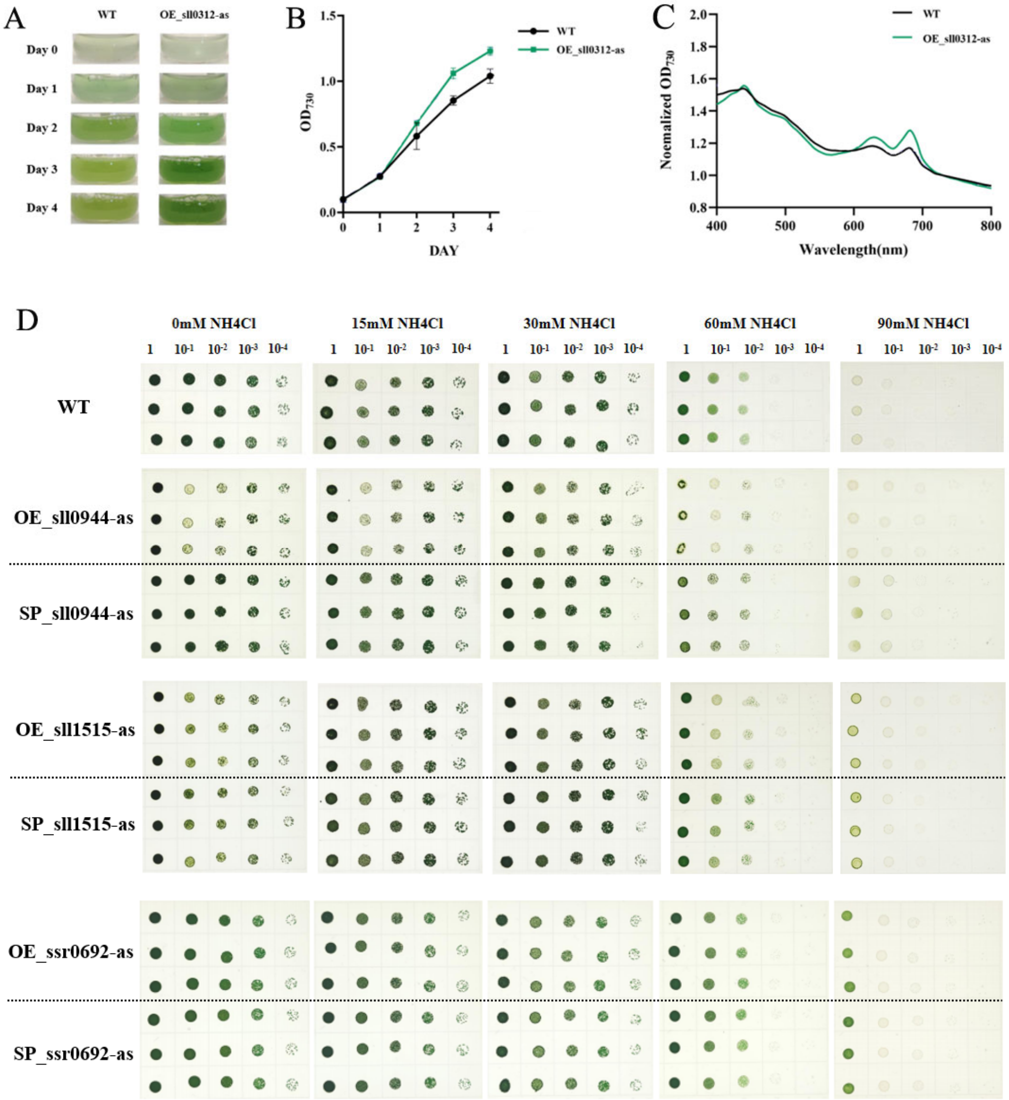

Supplementary Figure S5. Ammonium stress tolerance of overexpression or suppression strain of ammonium-responsive asRNAs. (A) Representative culture of OE_sll0312 compared with WT under ammonium stress. (B) Growth curve of OE_sll0312 and WT under 90 mM ammonium chloride. (C) The absorbance curve of OE_sll0312 and WT under 90 mM ammonium chloride. (D) No significant difference was observed in the drop-plate experiment among the overexpression or suppression strains of sll0944-as, sll1515-as, and ssr0692-as.

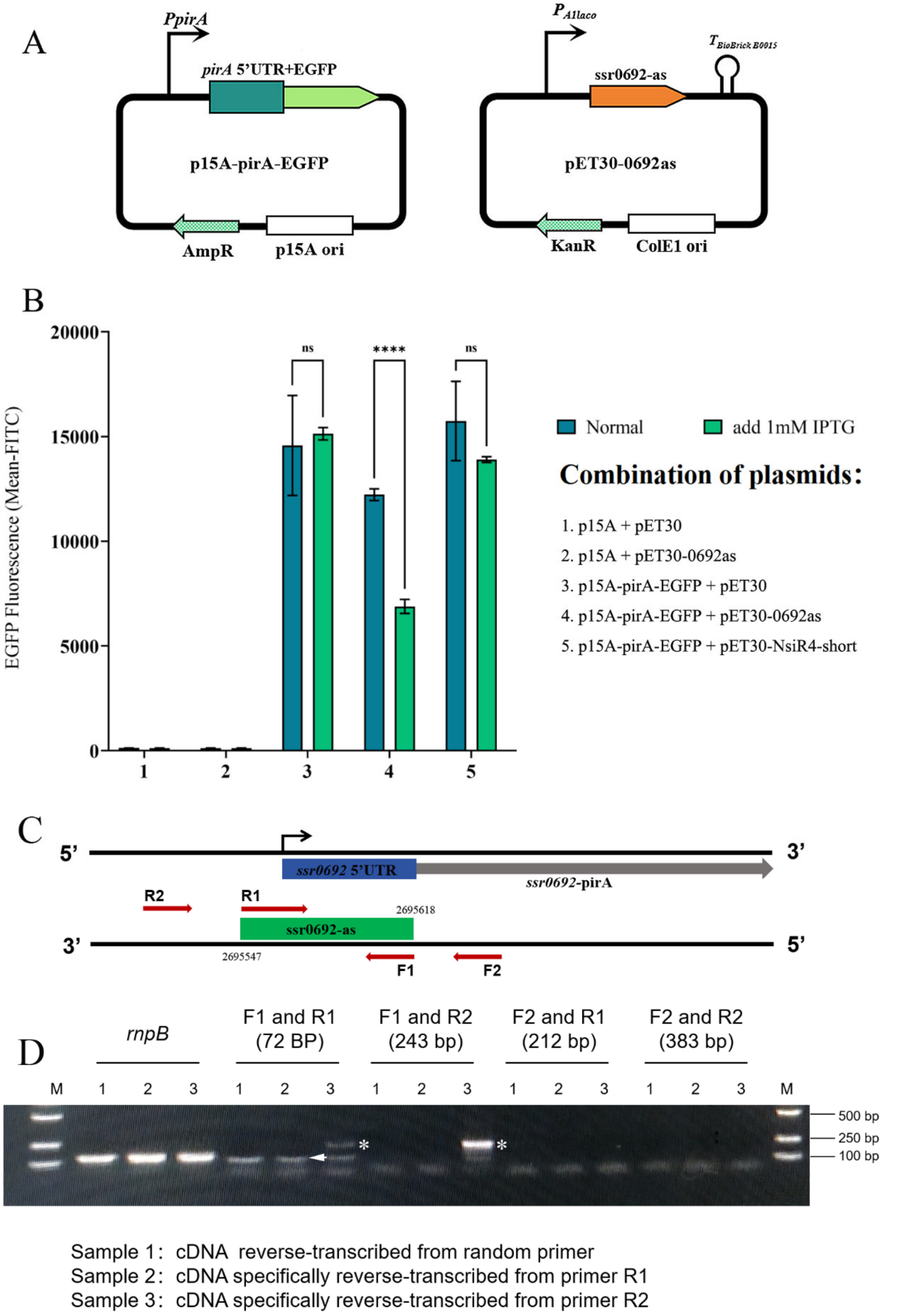

Supplementary Figure S6. Reporter assays verified the interaction between ssr0692-as and the 5'UTR of *pirA*. (A) Two plasmids for co-expression assays. The 5'UTR of *pirA* and its first 60nt were fused in frame with EGFP and expressed in p15A vector with medium-copy number replication origin p15A. The ssr692-as or the short version of NsiR4 was expressed

under the control of promoter PA1lacO-1 in pET30 vector with high copy number replication origin ColE1. (B) GFP fluorescence in TOP 10 with different plasmids combinations. The GFP translation and fluorescence were significantly suppressed by induction of ssr0692-as under IPTG, while no significant difference detected by empty pET30 vector without ssr0692-as. A short version of NsiR4 lack of the first seven nucleotide was also used as negative control. Data are the mean ± SD of three independent experiments, **** denotes $p<0.0001$ and ns indicates no significant difference. (C) Localization of primers used in D. (D) RT-PCR verification of ssr0692-as. Sequencing of the PCR products indicates that band with arrow was the correct ssr0692-as, while the two bands with * were nonspecific amplification from 23S ribosomal RNA.
