# Supplementary material for: Systematic and Functional Identification of Small Non-Coding RNAs Associated with Excess Ammonium Stress in Cyanobacterium Synechocystis sp. PCC 6803
Source: Int J Mol Sci. 2026 Jun 23;27(13):5667. doi: 10.3390/ijms27135667 (PMC13362535; doi:10.3390/ijms27135667)
Supplement: Supplementary file 1 [file ijms-27-05667-s001.zip › supplementary tables S2-S5.pdf]

Supplementary Table S2. Primers used for plasmid construction to generate asRNA overexpression or suppression strains.

Name	Sequence (5'→3')
sll1354-up-F	ttgcatgcctgcaggtcgacATGGCCCAATTTCGTTGGC
sll1354-up-R	tcttctctagaTGTTGGGGCACTGTGGGA
sll1354-down-F	taACCAGCTTTCGGGTAACGG
sll1354-down-R	atgaccatgattacgaattcTCATGGCTTGTCGGGTACGG
vector-F	GAATTCGTAATCATGGTCATAGCTG
vector-R	GTCGACCTGCAGGCATGC
psbA2-F	tgctcgatgagtttttctaaAACTGACTGACCACTGACCTTAAGAG
psbA2-R	ATAATTCCTTATGTATTTGTCGATGTTCA
$T_{biobrick\ B0015}$-F	GAGCTCCCAGGCATCAAATAAA
$T_{biobrick\ B0015}$-R	ccagcatcgcaaaataatttTATAAACGCAGAAAGGCC
Km^r-F	tccctttcaattctctaaccTCTAGAGAAGATCCTTTG
Km^r-R	TTAGAAAAACTCATCGAGCATCAAA
(+)sll0312-as-F	acataaggaattatggatccTGTTATGGCTAGTAG
(+)sll0312-as-R	tatttgatgcctgggagctcGGATTCCCGCCACCC
(-)sll0312-as-F	acataaggaattatggatccGGATTCCCGCCACCC
(-)sll0312-as-R	tatttgatgcctgggagctcTGTTATGGCTAGTAG
(+)sll0873-as-F	acataaggaattatggatccCCTGGGGTTTTCGAGAATGTT
(+)sll0873-as-R	tatttgatgcctgggagctcAAGACTTGATTGGGCTACTGTTTTATT
(-)sll0873-as-F	acataaggaattatggatccAAGACTTGATTGGGCTACTGTTTTAT
(-)sll0873-as-R	tatttgatgcctgggagctcCCTGGGGTTTTCGAGAATGTT
(+)sll0944-as-F	acataaggaattatggatccTAGGGACATTGGTAATGCGGG
(+)sll0944-as-R	tatttgatgcctgggagctcAGCCATCAGCCTTATTCTGTTGC
(-)sll0944-as-F	acataaggaattatggatccAGCCATCAGCCTTATTCTGTTGC
(-)sll0944-as-R	tatttgatgcctgggagctcTAGGGACATTGGTAATGCGGG
(+)sll1515-as-F	acataaggaattatggatccAATTTATGCCCTTAAAGTCA
(+)sll1515-as-R	tatttgatgcctgggagctcAAGGCGTGGCCATGAGCTAA
(-)sll1515-as-F	acataaggaattatggatccAAGGCGTGGCCATGAGCTAA
(-)sll1515-as-R	tatttgatgcctgggagctcAATTTATGCCCTTAAAGTCA
(+)slr1667-as-F	acataaggaattatggatccGTAGATAATGGTATCGCCAGCATC
(+)slr1667-as-R	tatttgatgcctgggagctcAAGTGCGGCTGGGGGAGT
(-)slr1667-as-F	acataaggaattatggatccAAGTGCGGCTGGGGGAGT
(-)slr1667-as-R	tatttgatgcctgggagctcGTAGATAATGGTATCGCCAGCATC
(+)ssr0692-as-F	acataaggaattatggatccACTTCTACCCTCCGAAAGGCA
(+)ssr0692-as-R	tatttgatgcctgggagctcACCTATAATTTTATGTTATGTTG
(-)ssr0692-as-F	acataaggaattatggatccACCTATAATTTTATGTTATGTTG
(-)ssr0692-as-R	tatttgatgcctgggagctcACTTCTACCCTCCGAAAGGCA

Plasmids were constructed by Gibson assembly using primers with lowercase sequences indicating the overlapping homology regions. (+) and (-) indicate primers for overexpression or suppression strains, respectively.

Supplementary Table S3. Primers used for plasmid construction to generate knockout strain of *sll0312*, *sll0873*, and *slr1667*.

Name	Sequence (5'→3')
KanR- F	TCAGAGAAGATCCTTTGATCTTTTCTA
KanR- R	TTAGAAAAACTCATCGAGCATCAAA
KO-vector-F	GGATCCTCTAGAGTCGACCTGCA
KO-vector-R	GAATTCACTGGCCGTCGTTTT
sll0312-up- F	AAACGACGGCCAGTGAATTCgagtcgggatctagttcagctttaa
sll0312-up- R	GATCAAAGGATCTTCTCTGAggattactgaattaagaactgctggt
sll0312-down- F	GATGCTCGATGAGTTTTTCTAAaattcccactcaatgtctgaatagtt
sll0312-down- R	AGGTCGACTCTAGAGGATCCgcgggttatgggttacatcg
sll0873-up- F	AAACGACGGCCAGTGAATTCaataatggcgatcgcgctc
sll0873-up- R	CAAAGGATCTTCTCTGAtttagctatagataaagaaaaaagactactgc
sll0873-down- F	GATGCTCGATGAGTTTTTCTAAcgaaccattggggtcgtca
sll0873-down- R	AGGTCGACTCTAGAGGATCCatctccaccaggggcaacc
slr1667-up- F	AAACGACGGCCAGTGAATTCtggccatgtgcaaggcat
slr1667-up- R	GATCAAAGGATCTTCTCTGAaatttgtcgcctccttcccg
slr1667-down- F	GCTCGATGAGTTTTTCTAAttaaattaagggaattggcgttc
slr1667-down- R	AGGTCGACTCTAGAGGATCCgtttttccctctgaagtcagattga

Supplementary Table S4. Primers for heterologous reporter assays.

Name	Sequence (5'→3')
pETDuet-1-F	CTGCTGCCACCGCTGAGC
pETDuet-1-R	TCTACGCCGGACGCATCG
PirA-F	CACGATGCGTCCGGCGTAGAAAACAACGCTCCTAATGTTT
PirA-R	TCACCATACGCAGGGTTTCTTTGTGGG
egfp-F	TGTTTAACTTTAAGAAGGAGATGGTGAGCAAGGGCGAGG
egfp-R	TTGCTCAGCGGTGGCAGCAGTTACTTGTACAGCTCGTCCA
p15A-ori-F	GAAAAGATCAAAGGATCTTCTTGAGATCGTTTTGGTCTGC
p15A-ori-R	AAGGCCGCGTTGCTGGCGTTTTTCCATAGGCTCCGCCC
pET-ori-F	AACGCCAGCAACGCGGC
pET-ori-R	GAAGATCCTTTGATCTTTTCTACGG
pET-30b-F	ATATACATATGCACCATCATCA
pET-30b-R	ATTGCGTTGCGCCTCCTTCT
0692-T7-F	CTGACCTAGACTTCTACCCTCCGAAAGGCA
0692-T7-R	ATGATGATGATGATGGTGCATATGTATATCGCAGAAAGGC
P_{A1laco}-F	AGGCGCAACGCAATTAATGTGAGTTAGCTCACTCATTA
P_{A1laco}-R	GGTAGAAGTCTAGGTCAGTCCTCCATAAACAT
NsiR4-F	TTTATGGAGGACTGACCTAG**AAGACAT**AAAGTCAATATCACCCTCCG
NsiR4-R	TATTTGATGCCTGGGAGCTCTAAAGGACTAATAAACTCTAAAAAGAAAGCC
NsiR4-F-Short	GTTTATGGAGGACTGACCTAGAAAGTCAATATCACCCTCCGATTG
NsiR4-R-Short	TATTTGATGCCTGGGAGCTCAAAAAGAAAGCCGCCACTGA

The first seven nucleotide absent in the short version of NsiR4 fragment was underlined.

Supplementary Table S5. Primers used in quantitative RT-PCR.

Name	Sequence (5'→3')
ncr0640-S	CGCAGTCACGAACTGGACAA
ncr0640-A	ACGCAATGCCTGAGAATGGT
ncr0690-S	GGCTATGGAAACCCGACAGA
ncr0690-A	TGGAAGGTACCGAAACCCCT
NsiR4-RT-F	AAGACATAAAGTCAATATCACCCTCCG
NsiR4-RT-R	AAAAAGAAAGCCGCCACTG
sll0312-RT-F	TGTTATGGCTAGTAGTAGG
sll0312-RT-R	ATAGAACGGATTCCCGCCAC
sll0873-RT-F	CCTGGGGTTTTCGAGAATGTT
sll0873-RT-R	AAGACTTGATTGGGCTACTGTTTTATT
sll0944-RT-F	TAGGGACATTGGTAATGCGGG
sll0944-RT-R	AGCCATCAGCCTTATTCTGTTGC
sll1321-as-S	AGGGTCCAGGTCAACAACTG
sll1321-as-A	TGGAAGCGGAAACGGTAGTG
sll1515-RT-F	AATTTATGCCCTTAAAGTCAAACTAGAT
sll1515-RT-R	AAGGCGTGGCCATGAGCT
slr0517-as-S	ATGGGGAAAGTCGCACAGTT
slr0517-as-A	CAGAGATCGGCGGTTACTGG
slr1102-as2-S	CTACGGTATCTCCGGAACGC
slr1102-as2-A	AGGCCTATGGCTTTGGCTTT
slr1667-RT-F	GTAGATAATGGTATCGCCAGCATC
slr1667-RT-R	AAGTGCGGCTGGGGGAGTGCTTAA
sRNA 4-S	GGCTATGGAAACCCGACAGA
sRNA 4-A	TGGAAGGTACCGAAACCCCT
ssr0692-as-F1	ACTTCTACCCTCCGAAAGGCA
ssr0692-as-R1	TACCTATAATTTTATGTTATGTTGAATGTAAATC
ssr0692-as-F2	CAGCTTCTGCTTCCAATTGAC
ssr0692-as-R2	TTATCGCACCGGACTTCCA
ssr3129-as-S	CAAGGTTCATGGTTGATGCGG
ssr3129-as-A	ACCGATGATTTGGCACCCAG
RT-rnpB-F	AACAGCAACCAGTAAACAGT
RT-rnpB-R	TTTACCGAGCCAGTACCTCT
